# Reliability of Ultrasound Measurements of the Median Nerve in Asymptomatic Subjects Using a Handheld Device

**DOI:** 10.3390/s24113444

**Published:** 2024-05-27

**Authors:** Abdulrahman M. Alfuraih, Rana Hussain Aldahlawi, Yomna S. Habib, Ahmed S. Alhowimel, Mohamed Abdelmohsen Bedewi

**Affiliations:** 1 Radiology and Medical Imaging Department, College of Applied Medical Sciences, Prince Sattam bin Abdulaziz University, Kharj 16278, Saudi Arabia; 2 Department of Radiological Sciences, College of Applied Medical Sciences, King Saud University, Riyadh 14511, Saudi Arabia; rdahlawi@ksu.edu.sa; 3 Department of Radiology, University Hospital, Prince Sattam bin Abdulaziz University, Kharj 16278, Saudi Arabia; y.habib@psau.edu.sa; 4 Department of Health and Rehabilitation Sciences, College of Applied Medical Sciences, Prince Sattam bin Abdulaziz University, Kharj 16278, Saudi Arabia; a.alhowimel@psau.edu.sa; 5 Department of Internal Medicine, College of Medicine, Prince Sattam bin Abdulaziz University, Kharj 16278, Saudi Arabia; m.bedewi@psau.edu.sa

**Keywords:** handheld ultrasound, median nerve, carpal tunnel syndrome, ultrasound reliability, intra-operator reproducibility, inter-operator reproducibility, diagnostic imaging, point-of-care ultrasound

## Abstract

This study investigated the reliability of measuring the median nerve cross-sectional area (CSA) at the carpal tunnel inlet using a handheld ultrasound device (HUD) compared to a standard ultrasound system, focusing on intra- and inter-operator reproducibility among novice and expert operators. Employing a prospective cross-sectional design, 37 asymptomatic adults were assessed using both devices, with measurements taken by an expert with over five years of experience and a novice with less than six months. The CSA was determined using manual tracing and ellipse methods, with reproducibility evaluated through intraclass correlation coefficients (ICCs) and agreement assessed via Bland–Altman plots. Results showed a high degree of agreement between the devices, with excellent intra-operator reproducibility (ICC > 0.80) for the expert, and moderate reproducibility for the novice (ICCs ranging from 0.539 to 0.841). Inter-operator reliability was generally moderate, indicating acceptable consistency across different experience levels. The study concludes that HUDs are comparable to standard ultrasound systems for assessing median nerve CSA in asymptomatic subjects, with both devices providing reliable measurements. This supports the use of HUDs in diverse clinical environments, particularly where access to traditional ultrasound is limited. Further research with a larger sample and symptomatic patients is recommended to validate these findings.

## 1. Introduction

Ultrasound has become a valuable tool in the assessment and diagnosis of carpal tunnel syndrome (CTS). The reliability of ultrasound measurements of the median nerve, which is often compressed in CTS, has been extensively studied and established [1]. This non-invasive imaging technique allows for the visualization of the nerve, and enables the measurement of its cross-sectional area (CSA), aiding in the detection of nerve compression and the severity of CTS.

While ultrasound, including the techniques evaluated in this study, provides valuable anatomical and functional information, it is most effective when used as a complement to clinical symptom evaluation [2]. Clinical assessments remain paramount in the initial diagnostic process for CTS, guiding further diagnostic testing. Following clinical evaluation, ultrasound serves as a non-invasive, accessible option that can help substantiate the clinical diagnosis before more invasive tests, like electrophysiology, are employed. This sequence ensures a comprehensive approach, integrating symptomatology with imaging and functional tests to achieve an accurate diagnosis.

Several studies have consistently reported excellent intra- and inter-operator reliability for ultrasound measurements of the median nerve [3,4]. This means that different operators can obtain consistent and accurate measurements, enhancing the reliability and validity of the results. These findings support the use of ultrasound as a reliable diagnostic tool for evaluating CTS.

In terms of diagnostic accuracy, ultrasound has shown promising results. A meta-analysis conducted by Fowler et al. [5] found that ultrasound had a sensitivity of 77.6% and a specificity of 86.8% in diagnosing CTS. These values indicate that ultrasound is capable of correctly identifying a significant proportion of CTS cases while also ruling out the condition in healthy individuals. As a result, ultrasound has the potential to serve as a first-line confirmatory test for CTS.

Recent research has further supported the use of ultrasound as an alternative diagnostic test for CTS. A meta-analysis conducted by Zaki et al. in 2022 [6] confirmed that ultrasound exhibits equivalent sensitivity and slightly greater specificity compared to nerve conduction studies and electromyography. Moreover, A recent meta-analysis on the cross-sectional areas of the median nerve in carpal tunnel syndrome (CTS) based on electrodiagnostic classification found mean values of 11.6 mm^2^ for mild CTS, 13.7 mm^2^ for moderate CTS, and 16.8 mm^2^ for severe CTS, with a total sample size of 2292 wrists across the severity levels [1]. This shows that ultrasound could provide accurate and reliable diagnostic information for CTS, potentially reducing the need for more invasive or costly procedures.

To overcome the limitations of cost and accessibility associated with traditional ultrasound machines, handheld ultrasound devices (HUDs) have gained popularity. These portable devices offer a more convenient and cost-effective option for conducting ultrasound examinations, including the evaluation of the median nerve in CTS. Studies focusing on HUDs have reported good overall agreement between handheld devices and standard ultrasound systems across various medical fields, including abdominal, obstetrics, and gynecology studies [7,8,9].

In musculoskeletal imaging, HUDs have also demonstrated their efficacy. A study by Falkowski [10] et al. found a 65% agreement between HUDs and standard ultrasound in this context. However, it is important to note that this study did not assess inter- or intra-observer variability, nor did it involve observers with different levels of experience. Therefore, further research is needed to understand the capabilities and limitations of HUDs in comparison to standard ultrasound machines.

While ultrasound has proven to be a reliable and accurate tool for assessing the median nerve and diagnosing CTS, the use of HUDs offers a promising alternative that can potentially improve accessibility and affordability. Further studies comparing the diagnostic performance of HUDs and standard ultrasound machines in imaging the cross-sectional area of the median nerve at the carpal tunnel inlet would provide valuable insights into the effectiveness of these devices for CTS diagnosis.

The objective of this study was to investigate the CSA of the median nerve at the carpal tunnel inlet, comparing between an HUD and a standard ultrasound system, in addition to determining the intra- and inter-operator reproducibility between a novice and an expert operator of medical ultrasound equipment.

## 2. Materials and Methods

### 2.1. Design

The study was designed as a prospective cross-sectional study that was conducted at the University Hospital of Prince Sattam bin Abdulaziz University, Saudi Arabia. Ethical approval was acquired from the research ethics committee at Prince Sattam bin Abdulaziz University (approval No: REC-HSD-116-2022). All volunteers provided their consent to participate.

### 2.2. Subjects

Asymptomatic adult subjects were invited to participate if they had none of the following exclusion criteria: under 18 years of age, experienced any discomfort such as pain, altered sensation, or weakness in the hand, wrist, or upper extremities in the past 12 months, or had any injuries to the spine, neck, back, or upper extremities. Exclusion was also applied to those with arthritis or a history of surgery or fractures in the upper extremities. To achieve a meaningful intraclass correlation coefficient (ICC) of at least 0.60, with a power of 80% (1 − β = 80) and a significance level of 0.05 (α = 0.05), a minimum of 20 cases was necessary, as indicated by a reference study [3].

### 2.3. Instruments and Operators

All participants were scanned initially using a standard ultrasound machine (EPIQ 7, Philips Healthcare, Bothell, Washington, USA) with a high-frequency 5–18 MHz linear array transducer (L18-5). This was followed by scanning them using an HUD (Butterfly iQ by Butterfly Network Inc., Guilford, Connecticut, USA). The Butterfly iQ HUD utilizes Capacitive Micromachined Ultrasonic Transducers (CMUTs) for transmitting and receiving ultrasonic signals, through a single probe that operates at frequencies between 1 and 10 MHz. The technical specifications of the two machines are listed in Table 1. The HUD was linked to a 12.9-inch iPad Pro (Apple Inc., Cupertino, CA, USA) to capture the images.

An expert examiner (operator A), with more than 5 years of musculoskeletal ultrasound experience, scanned both wrists of each participant. To evaluate inter-operator reproducibility, a secondary novice operator (operator B), with less than 6 months of ultrasound experience, acquired secondary measurements. Each operator acquired the measurements independently and was blinded to the other operator’s images.

Participants were seated comfortably in a chair, with their forearms placed on an examination table and elbows bent at about 90 degrees. Their wrists were positioned neutrally, and their fingers extended straight. Both the left and right sides of each participant were assessed. The transducer was positioned transversely to the forearm at the level of the trapezium–hamate [3,4]. It was applied with no additional pressure beyond the probe’s weight to avoid distorting the tunnel or the median nerve.

### 2.4. Image Acquisition

**Figure 1:**
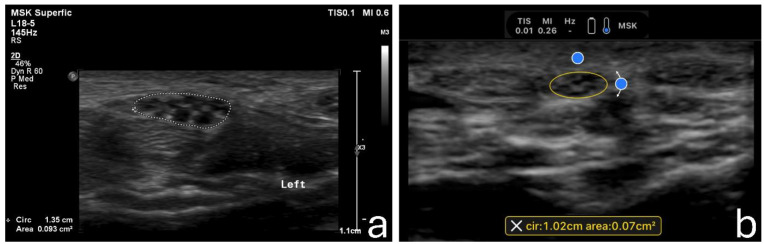
Examples of the acquired images using the standard machine employing the manual tracing measurement technique (**a**) and using the handheld device employing the ellipse measurement technique (**b**).

The primary measurement was the cross-sectional area (CSA) of the median nerve at the wrist level. In each wrist, three repeated measurements were acquired. For each measurement, a new image was captured after lifting and reapplying the transducer to ensure true replication of the actual process. The hyperechoic inner border of the epineurium was used as the reference point when measuring the CSA [3,4]. Two measurement techniques were employed in the standard machine, manual tracing and an electronic ellipse function. The latter was the only suitable measurement technique for the HUD. Examples of the techniques are shown in Figure 1.

### 2.5. Statistical Analysis

To investigate the reproducibility of the measurements, intraclass correlation coefficients (ICCs) were computed. The results were interpreted as follows: 0.00–0.20, ‘poor agreement’; 0.21–0.40, ‘fair agreement’; 0.41–0.60, ‘moderate agreement’; 0.61–0.80, ‘substantial agreement’; and >0.80, ‘almost perfect agreement’ [11]. Further, Bland–Altman plots were constructed to analyze the agreement between the two systems. The 95% limits of agreement (LoAs) were calculated as the mean difference plus and minus 1.96 of the standard deviation (SD) of the differences [12]. All analyses were done using SPSS 29 Statistics Package (SPSS Inc., Chicago, IL, USA).

## 3. Results

**Figure 2:**
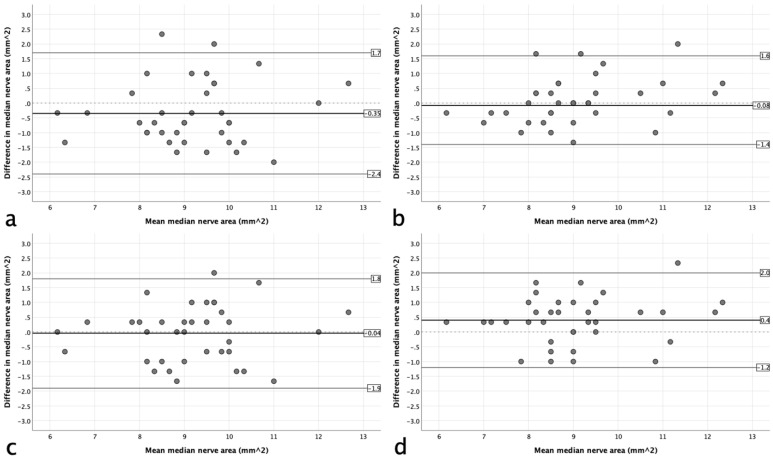
Bland–Altman plots of the agreement between the handheld device and standard system for use of the tracing method (**a**,**b**) and ellipse method (**c**,**d**) on the right (**a**,**c**) and left sides (**b**,**d**).

A total of 37 participants (22 females and 15 males) aged 20–56 years (mean [SD] 34 years [7.9]) were recruited for the study. The mean (SD) BMI was 24.8 (3.4). The descriptive statistics for the median nerve area are presented in Table 2. The table shows that using the tracing method, the median nerve area had a mean difference between the HUD and the standard system of 0.35 mm^2^ (3.80%) on the right side and a negligible difference on the left side (0.9%). In contrast, the ellipse method had a mean difference of −0.4 mm^2^ (−4.4%) on the left side and a negligible difference on the left side (0.5%). The agreement results are illustrated graphically on the Bland–Altman plots in Figure 2. They showed no evidence of systematic bias based on the degree of the mean area.

As for the reproducibility of the measurements, the expert operator (A) had almost perfect intra-operator reproducibility, as demonstrated in Table 3. The novice operator, however, had a fair-to-moderate agreement, with coefficients ranging from 0.539 to 0.841.

## 4. Discussion

The aim of this study was to investigate the cross-sectional area (CSA) of the median nerve at the carpal tunnel inlet using both a handheld ultrasound device (HUD) and a standard ultrasound system. Additionally, the study aimed to evaluate the reproducibility of measurements by comparing the performance of a novice operator and an expert operator in medical ultrasound. Overall, the results suggest that HUDs could reliably substitute for traditional systems in settings where standard ultrasound equipment is not available. The expert operator achieved excellent intra-operator reproducibility, highlighting the importance of experience in obtaining consistent ultrasound measurements. Although the novice operator’s reproducibility ranged from fair to moderate, this variability underscores the necessity for thorough training to ensure reliability across different operators. Inter-operator reliability, while varied, was generally acceptable, suggesting that standardized training and calibration could enhance consistency.

The results showed an almost perfect agreement between the handheld device and the conventional machine when using the ellipse method to measure the median nerve area in both the right and left hands. Even when comparing the tracing method in the conventional machine with the ellipse method in the HUD, the difference was minimal. These findings are consistent with more recent results from Zardi et al. [13].

Furthermore, the current results are superior to the findings reported by Falkowski et al. [10], where they reported only a 65% agreement in diagnosis between the HUD and conventional ultrasound. However, it should be noted that the authors emphasized that this substantial percentage of disagreement did not impact clinical management. The potential reason for the lower agreement results could be the different anatomical parts studied (shoulder, elbow, knee, and calf) compared to ours.

Regarding reproducibility, the results showed that even when a non-experienced operator used the HUD, fair-to-moderate agreement could be achieved in measuring the cross-sectional area (CSA) of the median nerve. These findings are consistent with the results reported in a previous study [14], where they concluded that median nerve CSA measurements using direct and indirect methods demonstrated good reproducibility with high intra-observer correlations and slightly lower but still good inter-observer correlations.

When discussing the measurement technique, it should be noted that, to date, there is no standardized protocol for measuring the CSA of the median nerve [15]. In our study, the HUD software (Version 2.15.0) was not programmed to perform the manual tracing measurement, which could be suggested as a more reproducible, and certainly more popular, technique compared to the ellipse technique [16]. Others, however, showed evidence claiming a similar performance between the two techniques [17]. The problem with the electronic ellipse measurement technique is the potential bias when the nerve becomes less elliptical and more ovoid [18].

The results obtained in the current study align with the findings reported by Bueno-Gracia et al. [3], where they found excellent reliability for carpal tunnel and median nerve measurements in asymptomatic subjects. This indicates that ultrasound is a reliable tool for these measurements in asymptomatic individuals.

The HUD provides a cost-effective alternative for implementing ultrasound in remote point-of-care environments. It serves as a portable, non-invasive, and radiation-free bedside assessment tool, which is widely familiar to primary care and specialized clinicians [8]. In clinical scenarios where transporting a patient to the ultrasound department is not possible or the conventional ultrasound machine is less accessible, the use of HUD ultrasound can be advantageous. Understanding the advantages and limitations of portable handheld ultrasound devices will help identify the appropriate areas where specific types of ultrasound equipment can be utilized. Recent advancements in ultrasound imaging technologies, such as microvascular imaging, have shown promise in enhancing the diagnosis of CTS. Previous studies highlighted the sensitivity of microvascular imaging in demonstrating intraneural vascularity, surpassing conventional Doppler imaging [19,20]. Additionally, microvascular imaging has proven beneficial as a diagnostic tool for CTS, showing a correlation with the degree of neuropathy and nerve compression identified in nerve conduction studies [21]. These findings underscore the potential of integrating advanced ultrasound techniques with traditional CSA measurements to provide a more nuanced and effective diagnostic approach for CTS, enhancing both detection and grading of the condition. One strength of this study is that the operators were blinded to each other’s images, ensuring unbiased results, unlike several previous studies cited in Rykkje et al.‘s systematic review [7]. Nevertheless, it is important to acknowledge that this study has some limitations. Although a sample size of 20 cases was sufficient to ensure reliable results, as indicated by a reference study, the small sample size in the current study may limit the generalizability of the findings to the larger population. Additionally, to maintain consistency in data collection, our study subjects consisted of asymptomatic volunteers, which may not represent the clinical demographic characteristics of patients affected by CTS. Nevertheless, the primary focus of this study was on comparing the agreement between HUDs and conventional ultrasound systems, rather than identifying differences within a patient group. To validate this, future studies should aim at studying the clinical effectiveness and diagnostic accuracy of HUDs in patients with CTS. Finally, the study’s inter-rater reliability analysis may be constrained by the inclusion of only two operators, one experienced and one novice. This small sample of operators might not fully represent the range of skills and experiences typically encountered in clinical practice, potentially limiting the generalizability of our findings to broader settings. Future studies should aim to include a larger, more diverse group of operators, encompassing multiple levels of experience and expertise. A block design involving several experienced and novice operators could provide a more robust analysis of inter-rater reliability.

## 5. Conclusions

The study demonstrated that handheld ultrasound devices (HUDs) provide a high level of agreement with standard ultrasound systems when measuring the cross-sectional area (CSA) of the median nerve at the carpal tunnel inlet, indicating minimal differences in measurements between the two devices. The findings underscore the utility of HUDs in enhancing the accessibility of ultrasound diagnostics, particularly in resource-limited environments. Given their portability and ease of use, HUDs could significantly expand the reach of point-of-care diagnostics, potentially improving early diagnosis and patient outcomes in primary care settings. Overall, this study supports the viability of HUDs as effective tools for the non-invasive assessment of the median nerve, offering a promising alternative for widespread clinical use.

## Figures and Tables

**Table 1:** The technical specifications of the two machines.

	EPIQ 7, Philips Healthcare	Butterfly iQ, Butterfly Network Inc.
**Transducer technology**	Broadband 288 piezoelectric elements	9000 = element Capacitive Micromachined Ultrasonic Transducer (CMUT)
**Transducer type**	Linear transducer	Multi-modal 2-D array
**Frequency range (MHz)**	18–5	10–1
**Weight (kg)**	104.3	0.3
**Dimensions (mm)**	606 × 1500 × 1009	163 × 56 × 35

**Table 2:** The mean area of the median nerve and differences between the standard system and the handheld device.

	Standard (Tracing) (SD)	Standard (Ellipse) (SD)	Handheld (SD)	Handheld versus Standard (Tracing)			Handheld versus Standard (Ellipse)		
				Mean Difference (%)	Upper and Lower 95% LoAs	*p*-Value *	Mean Difference (%)	Upper and Lower 95% LoAs	*p*-Value *
**Right**	8.9 ± 1.4	9.3 ± 1.4	9.3 ± 1.3	0.35 (3.8%)	−2.4, 1.7	0.026	0.04 (0.5%)	−1.9, 1.8	0.386
**Left**	9.0 ± 1.5	9.4 ± 1.4	9.0 ± 1.3	0.08 (0.9%)	−1.4, 1.6	0.265	−0.4 (−4.4%)	−1.2, 2.0	0.002

All measurements are presented in mm^2^ unless otherwise indicated. * *p*-values were calculated using paired-samples *t*-test.

**Table 3:** Intraclass correlation coefficients for intra- and inter-operator reproducibility results.

	Right			Left		
	Standard (Tracing)	Standard (Ellipse)	Handheld	Standard (Tracing)	Standard (Ellipse)	Handheld
**Operator A reproducibility**	0.901 (0.830, 0.946)	0.894 (0.815, 0.943)	0.906 (0.838, 0.948)	0.921 (0.864, 0.957)	0.916 (0.855, 0.954)	0.926 (0.873, 0.960)
**Operator B reproducibility**	0.703 (0.128, 0.920)	-	0.539 (0.478, 0.880)	0.801 (0.391, 0.947)	-	0.841 (0.542, 0.957)
**Inter-operator reproducibility**	0.572 (0.283, 0.884)	-	0.714 (0.150, 0.930)	0.505 (0.297, 0.574)	-	0.674 (0.099, 0.915)

All results are presented as ICC (95% confidence interval).

## Data Availability

The data can be made available upon reasonable request to the corresponding author.
